# SNPs in cytochromes P450 catalyzing cholesterol degradation in brain are associated with Parkinson’s disease

**DOI:** 10.3389/fphar.2024.1477009

**Published:** 2024-09-30

**Authors:** Polina Petkova-Kirova, Anastasia Kolchina, Stephan Baas, Gudrun Wagenpfeil, Marcus Michael Unger, Julia Maria Schulze-Hentrich, Rita Bernhardt

**Affiliations:** ^1^ Institut für Biochemie, Fachbereich Biologie, Naturwissenschaftlich-Technische Fakultät, Universität des Saarlandes, Saarbrücken, Germany; ^2^ Institute of Neurobiology, Bulgarian Academy of Sciences, Sofia, Bulgaria; ^3^ Department of Genetics/Epigenetics, Faculty NT, Saarland University, Saarbrücken, Germany; ^4^ SHG Kliniken, Saarbrücken, Germany; ^5^ Institut für Medizinische Biometrie, Epidemiologie und Medizinische Informatik, Universität des Saarlandes, Homburg, Germany; ^6^ Klinik für Neurologie, SHG Kliniken Sonnenberg, Saarbrücken, Germany

**Keywords:** Parkinson's disease, cytochromes P450, CYP46A1, CYP27A1, CYP7B1, CYP39A1, cholesterol

## Abstract

Besides being an essential structural component of plasma membranes and the precursor of many functional compounds and signaling molecules, cholesterol was also proposed to play a role in the etiology and/or manifestation of Parkinson’s disease (PD). However, so far systematic investigations on the role of cholesterol and its metabolites present in the brain for the etiology of PD are missing. Here, we investigate for the first time the association of PD with SNPs in the genes of four cytochromes P450 (P450), CYP46A1, CYP39A1, CYP27A1 and CYP7B1, which are critical for the degradation of cholesterol in the brain. Analyzing 1,349 individuals from the PPMI data base, we found 24 SNPs in these four genes, which are significantly over- or under-represented in patients suffering from idiopathic PD (IPD). Studying each of the 362 IPD patients individually, we found that most patients (45%) showed only one associated SNP in one of the four P450 genes, while 31% displayed two associated SNPs and 18% three associated SNPs. The occurrence of some associated SNPs is in the same order of magnitude as SNPs in the GBA (beta-glucocerebrosidase) and thus might reflect a genetic predisposition for PD. As all 24 SNPs were located in introns and 3′ untranslated regions, we evaluated the prospective regulatory impact of the surrounding genomic regions by using transcriptome and epigenome data from the Foundational Data Initiative for Parkinson Disease (FOUNDIN-PD). FOUNDIN-PD provides gene expression, open chromatin and DNA methylation data in a cohort of 89 induced pluripotent stem cell (iPSC) lines differentiated to dopaminergic (DA) neurons derived from people in the PPMI study. Indeed, two of the 24 SNPs, one in CYP7B1 (rs118111353) and the other one in CYP27A1 (rs74446825), were localized within a region of open chromatin in differentiated neurons. Interestingly, all iPSC lines with open chromatin in rs118111353 showed the reference allele. As all four P450, CYP46A1, CYP39A1, CYP27A1 and CYP7B1, are expressed in dopaminergic neurons, we discuss further functional studies to connect SNPs in regulatory regions with gene expression levels. Finally, potential possibilities for personalized therapeutic treatment of patients with SNPs in the four investigated P450 are discussed.

## Introduction

Parkinson’s disease (PD) which was first described by James Parkinson in 1817 (Simon et al., 2020) is the second most common neurodegenerative disorder. From 1990 to 2015 the number of people suffering from PD has increased from 2.6 to 6.3 million and a further increase to approximately 17.5 million is being expected until 2040 (Dorsey et al., 2018). This is not only a tremendous clinical challenge but also an emerging social problem. PD is characterized by progressive degeneration of the *Substantia nigra pars compacta* leading to a loss of dopaminergic neurons. Unfortunately, there is a lack in understanding the etiology of PD and, therefore, causative treatments. Recently, the effect of SNPs in the genes of the cytochrome P450 (P450) family has been shown to be related to PD (Hartz et al., 2022). Cytochromes P450 are hemoproteins catalyzing the hydroxylation of aliphatic and aromatic compounds via activation of molecular oxygen and mono-oxygenase-type cleavage of this latter molecule. Electrons necessary for the latter reaction are provided by NAD(P)H and transported via various types of redox chains (Hannemann et al., 2007). P450 are able to hydroxylate a broad variety of substrates reaching from alkanes and fatty acids to xenobiotics, sterols and complex antibiotics. In humans they are involved in the biotransformation of drugs and xenobiotics, in the metabolism of fatty acids and eicosanoids, the metabolism of vitamins, the biosynthesis and degradation of cholesterol, the biosynthesis of steroid hormones from cholesterol as well as in so far unknown functions (Guengerich, 2017). Analysis of the over- or under-representation of single nucleotide polymorphisms (SNPs) in the genes of all 57 human P450 as well as their three redox partners revealed that in 26 out of 57 P450 as well as in two out of the three redox partners SNPs with a very high over-representation (odds ratio of >5) were found in PD patients compared to healthy controls. Analyzing the function of the P450 with altered SNPs pointed especially at those P450 genes which displayed a prominent role in the biotransformation of xenobiotics, in the immune response and in cholesterol degradation in the brain (Hartz et al., 2022). Moreover, when considering the effect of SNPs in the genes of the P450 family to the manifestation of PD symptoms in patients with a genetic predisposition, it was shown that again cholesterol degradation was playing a critical role for the shift from people possessing a genetic predisposition but showing no symptoms of PD (GUN) to patients with the predisposition and showing symptoms (GPD patients) (Petkova-Kirova et al., 2023).

**FIGURE 1:**
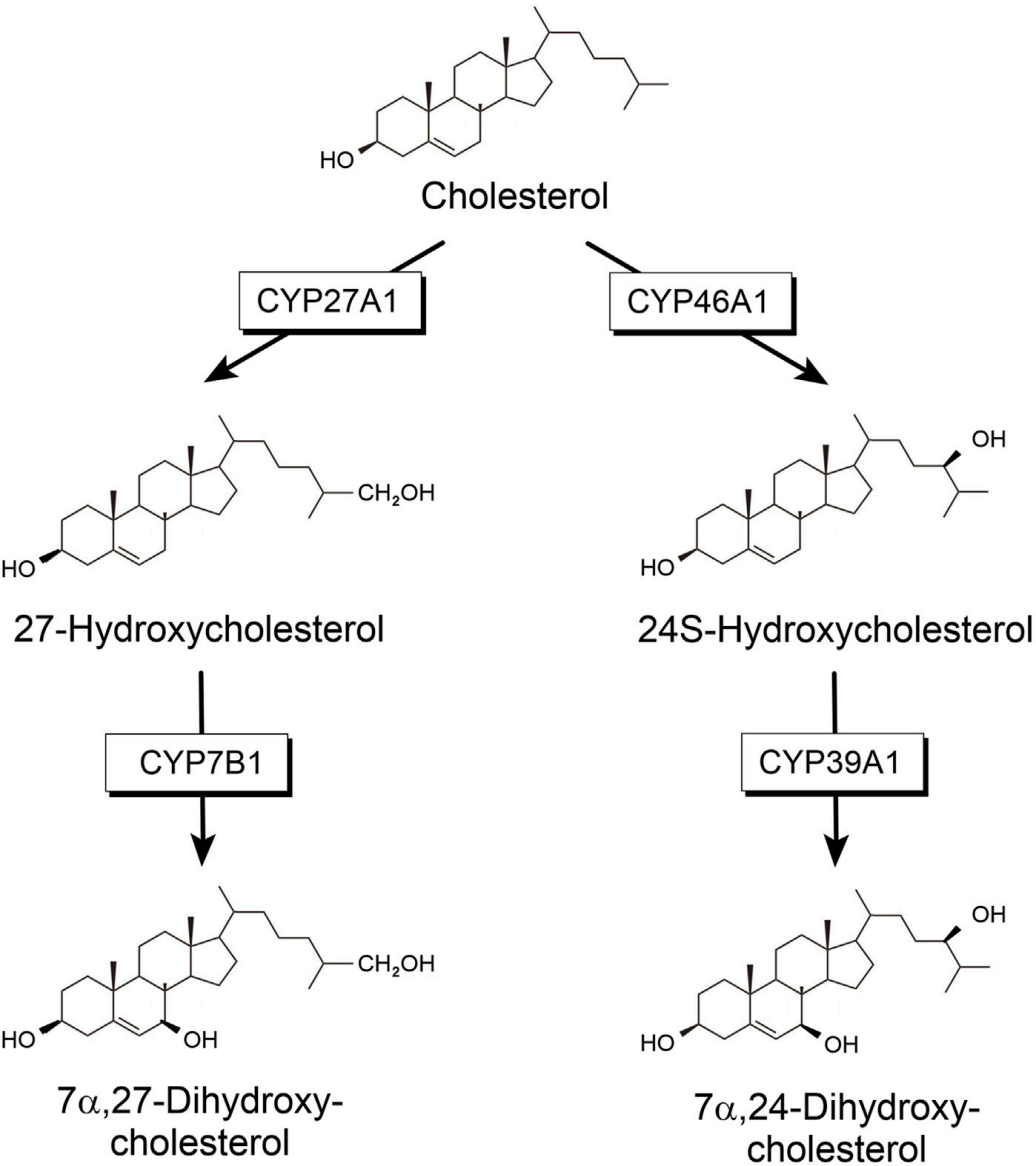
Cholesterol degradation.

Cholesterol is an important molecule mostly known for its role in cardiovascular disease. However, it seemingly has more functions in human metabolism. It is a vital component of cell membranes in vertebrates, where it regulates membrane fluidity. Furthermore, it is the precursor of all steroid hormones, which fulfill important regulatory functions in the metabolism (Bernhardt and Neunzig, 2021). About 23% of unesterified cholesterol can be found in the central nervous system (CNS) although it accounts for only 2% of the body mass (Dietschy and Turley, 2004). Despite its high content in brain, its turnover is very slow. The flux across the brain is only approximately 0.9% as rapid as the turnover in the whole body (Dietschy and Turley, 2001). The blood-brain barrier is impermeable for cholesterol and, therefore, cholesterol must be synthesized locally in the brain (Bjorkhem and Meaney, 2004). Consequently, cholesterol homeostasis in the brain reflects the balance between *in-situ* biosynthesis and elimination. To be exported from the brain, cholesterol needs to be modified into a more soluble compound, which can pass the blood-brain barrier. Locally synthesized cholesterol has been demonstrated to be hydroxylated and further metabolized mainly by steroid hydroxylases. Four steroid-hydroxylating P450 are involved in the degradation of cholesterol synthesized in the brain: CYP46A1, CYP39A1, CYP27A1 and CYP7B1 (Figure 1). CYP27A1 catalyzing hydroxylation in position 27 was found to be expressed mainly in the liver but also in many other tissues including brain. In addition, 24-hydroxycholesterol has been described as the main steroid to be excreted from the brain. This reaction was shown to be catalyzed by CYP46A1, which is expressed mainly in the brain and to a much lower extent in some other organs and tissues (Bjorkhem et al., 1999; Lund et al., 1999). When looking at further steps of cholesterol degradation in the brain, it turns out that CYP39A1, which is mainly expressed in the liver, but also found in the brain, catalyzes further hydroxylation of 24-hydroxycholesterol to produce 7α, 24-dihydroxycholesterol (Figure 1). On the other hand, 27-hydroxycholesterol can be further modified to 7α, 27-dihydroxycholesterol by CYP7B1, which is also expressed in the brain (Stiles et al., 2009). Final degradation of cholesterol to bile acids requires the participation of another P450, CYP8B1, as well as again CYP27A1.

It has been shown that defects in the biosynthesis or degradation of cholesterol in brain lead to neurodegeneration (Dietschy and Turley, 2001). Thus, CYP46A1 being involved in the efflux of cholesterol from the brain as well as in the activation of cholesterol biosynthesis in this organ, turns out to be a crucial player in the manifestation of various neurological diseases such as Alzheimer’s disease. Moreover, it was demonstrated in mouse models that modulation of CYP46A1 activity also affects other diseases such as Huntington’s, Machado-Joseph (MJD) and Nieman-Pick type C diseases as well as amyotrophic lateral sclerosis, epilepsy and glioblastoma (Pikuleva and Cartier, 2021).

Very recently, the potential two-fold role of cholesterol in Parkinson’s disease has been reviewed (Alrouji et al., 2023). The authors summarized its role, on one hand, in the deregulation of ion channels and receptors leading to a neuroprotective effect on this disease, and, on the other hand, its role in neuroinflammation via the release of pro-inflammatory cytokines. In addition, co-localization and association of cholesterol with α-synuclein has been described (Fortin et al., 2004). Elevated levels of cholesterol were shown to accelerate α-synuclein fibrilization (Bosco et al., 2006) and to induce degeneration of dopaminergic neurons in the *S. nigra pars compacta* (Hu et al., 2008; Zhaliazka et al., 2024). Although there are indications that α-synuclein and the formation of α-synuclein oligomers and fibrils play a critical role in neurotoxicity (Singleton et al., 2003), the detailed mechanism of this process is far from being understood. The role of enzymes being involved in cholesterol degradation and their association with PD is unclear as well.

Therefore, we decided to analyze the P450 involved in the degradation and efflux of cholesterol from the brain to get a deeper understanding of this process and its possible impact on the etiology of PD. We concentrated mostly on patients with idiopathic origin of PD (IPD patients), since they comprise the largest group of PD patients (approximately 85%). We have demonstrated that SNPs in four P450, CYP46A1, CYP39A1, CYP27A1 and CYP7B1, which are involved in cholesterol degradation in the brain are associated with the occurrence of IPD. Since all SNPs are located in regulatory regions, we also studied gene expression, open chromatin and DNA methylation connected to these SNPs.

## Materials and methods

Information for the genetic variants and statistical analysis of the occurrence of SNPs in the four P450 (CYP46A1, CYP39A1, CYP27A1 and CYP7B1) involved in cholesterol degradation was obtained as previously described (Hartz et al., 2022). Data used in the preparation of this article (Tier 1) were obtained on 27 August 2020 from the Parkinson’s Progression Markers Initiative (PPMI) database (https://www.ppmi-info.org/access-data-specimens/download-data, RRID: SCR_006431). For up-to-date information on the study, visit http://www.ppmi-info.org. Briefly, whole genome sequencing (WGS) VCF files were obtained from the Parkinson’s Progression Markers Initiative database for the four P450 and genetic variants were identified. Thе variants were annotated with SnpEff 5.0 and SnpSift 5.0, further processed with the R programming language and separated into groups (IPD and HC based on clinical diagnosis), in which groups further assessment, as detailed in Results, was performed. Altogether 362 IPD patients and 193 HC (healthy controls) were included into our analyses. IBM-SPSS Version 26 and 27 were used for statistical analysis. The reported *p* values are raw, two-sided, without an adjustment and the significance level set at 0.05. Risk factors are assessed using logistic regression and reported as odds ratios (OR) with 95% confidence intervals (CI). The ORs are calculated as follows: (number of IPD patients with a SNP/number of HC with a SNP)/(number of IPD patients without a SNP/number of HC without a SNP) and if OR is above 1, then it appears to be over-represented in IPD patients compared to HC, whereas if OR is below 1, it appears to be under-represented in IPD patients. We also make use of a comprehensive data set from the Foundational Data Initiative for Parkinson Disease (FOUNDIN-PD) (Bressan et al., 2023). From a large series of 95 iPSC lines derived from subjects within the PPMI database and driven to a DA neuronal cell type, bulk ATAC-Seq data profiling open chromatin, EPIC array data charting DNA methylation as well as bulk RNA-seq data measuring gene expression changes were available. In detail, 94 ATAC-seq samples are provided within FOUNDIN-PD, of which 5 were technical replicates, resulting in 89 samples to investigate. Here, we focused on the last timepoint of differentiation as it represents the differentiated dopaminergic neurons. Using the IDs of the samples, we matched metadata such as sex, age and disease status, obtained from tables provided by PPMI. Positions of open chromatin from the ATAC-seq data were overlapped with positions of the SNPs of interest. For each SNP within open chromatin, the number of samples with the reference or the alternative allele were calculated based on data from PPMI.

Similarly, for the EPIC array data from day 65, for all SNPs of interest, the distance to the closest CpG site was identified. DNA methylation levels between healthy controls and IPD patients were compared for those CpG sites, and the underlying allele color-coded.

Finally, expression levels visualized as RPKMs (Reads Per Kilobase per Million mapped reads) of CYP46A1, CYP39A1, CYP27A1 and CYP7B1 were compared in bulk RNA-seq samples of healthy controls and PD patients at day 65. Based on single-cell RNA-seq data from FOUNDIN-PD, median coverage in individual cell types were related at day 65.

## Results

### Recalculation of OR values of SNPs in line with a new reference genotype

As already mentioned by (Hartz et al., 2022), in a few cases the SNPs in individual P450 are not found with highest frequency in the studied PD individuals from the PPMI database but in the reference genome (e.g genotype 1/1 instead of 0/0 seems to represent the reference position). When looking at the SNPs found in the four P450 involved in cholesterol degradation (Figure 1) in IPD patients, it turns out that the above issue affects six SNPs in CYP46A1, CYP7B1 as well as CYP39A1 (namely: rs4905883 (genotypes 0/1 and 1/1), chr14:99713865_CAAAAA/CAAAAAAA (genotype 1/2, etc.), chr8:64607398_TAAA/TA (genotype 0/2, etc. and 2/2, etc.), chr6:46616160_CTTTT/CT (for genotype 1/1), rs3799866 (for genotype 0/1) and rs55887439 (for genotypes 0/1 and 0/2, etc.), marked in *italics* in Table 1A). We, therefore, re-calculated the OR values and confidence intervals of those SNPs taking into account the new reference genotype at the corresponding position. This resulted in four SNPs displaying significant OR values when comparing IPD and HC (rs4905883 (genotype 0/0), chr14:99713865_CAAAAA/CAAAAAAA (genotype 1/2, etc., note the change in OR from 2.29 to 2.37), chr8:64607398_TAAA/TA (genotype 0/0), rs55887439 (genotype 0/0), while two SNPs (chr6:46616160_CTTTT/CT, rs3799866) did not show significant ORs for any genotype (Table 1B). Taken together and as shown in Table 1B, 24 SNPs of the investigated four P450 were significantly over- or under-represented when comparing IPD patients and HC.

**TABLE 1A:** Summary of all significant single nucleotide polymorphisms (SNPs) in cytochromes P450 CYP7B1, CYP27A1, CYP39A1 and CYP46A1 when comparing HC and IPD patients. In **bold** are the SNPs that come significant when comparing GPD and HC patients as well; in *italics* are the SNPs that come with a reference genotype other than 0/0. 1/2, etc. refers to genotypes 1/2, 1/3, 1/4, 1/5, 1/6, 2/3, 2/4, 2/5, 2/6, 3/4, 3/5, 3/6, 4/5, 4/6, 5/6; 0/2, etc. refers to genotypes 0/2, 0/3, 0/4, 0/5, 0/6; 2/2, etc. refers to genotypes 2/2, 3/3, 4/4, 5/5, 6/6.

	SNP	Genotype	OR	95% CI		*p*-value
				Lower	Upper	
**CYP46A1**	*rs4905883*	0/1	10.95	1.23	97.02	0.032
	*rs4905883*	1/1	9.27	1.07	80.08	0.043
	chr14:99722645_G/*	1/1	1.85	1.07	3.21	0.027
	*chr14:99713865_CAAAAA/CAAAAAAA*	1/2, etc.	2.29	1.26	4.16	0.007
**CYP7B1**	rs2356986	0/1	1.47	1.02	2.14	0.040
	rs2356985	0/1	1.48	1.02	2.15	0.037
	rs2884074	0/1	1.48	1.02	2.15	0.037
	rs118111353	0/1	3.51	1.02	12.01	0.046
	**rs16931331**	1/1	4.43	1.00	19.59	0.049
	**rs16931334**	1/1	4.43	1.00	19.59	0.049
	***chr8:64607398_TAAA/TA***	0/2, etc.	0.26	0.07	0.90	0.034
	***chr8:64607398_TAAA/TA***	2/2, etc.	0.24	0.07	0.84	0.025
	rs117732106	0/1	0.36	0.13	0.96	0.041
	rs4477068	0/1	0.36	0.13	0.96	0.041
**CYP39A1**	rs200377987	0/1	3.51	1.02	12.01	0.046
	rs188027691	0/1	3.51	1.02	12.01	0.046
	rs115993944	0/1	3.51	1.02	12.01	0.046
	rs3799865	0/1	3.51	1.02	12.01	0.046
	rs34289054	0/2, etc.	0.43	0.18	0.99	0.048
	*chr6:46616160_CTTTT/CT*	1/1	0.49	0.25	0.96	0.038
	chr6:46650484_CTTTT/CTTTTTTT	1/1	0.36	0.16	0.80	0.013
	***rs3799866***	0/1	0.68	0.46	1.00	0.04982
	***rs55887439***	0/1	0.53	0.29	0.98	0.043
	*rs55887439*	0/2, etc.	0.52	0.32	0.86	0.011
**CYP27A1**	**rs692303**	0/1	0.19	0.05	0.74	0.016
	**rs73991002**	0/1	0.13	0.03	0.61	0.010
	rs692258	0/1	0.22	0.06	0.87	0.031
	rs692290	0/1	0.22	0.06	0.87	0.031
	**rs74446825**	0/1	0.54	0.31	0.94	0.029

**TABLE 1B:** Summary of all significant single nucleotide polymorphisms (SNPs) in the four P450 genes CYP7B1, CYP27A1, CYP39A1 and CYP46A1 involved in the degradation of cholesterol when comparing HC and IPD patients. In some cases, the OR values were recalculated compared with data given in (Hartz et al., 2022) considering that the reference genotype should be the one that occurs most abundantly in healthy people. In *italics* are the SNPs that come different compared to the same SNPs when 0/0 was taken as a control genotype (Table 1A). Note that SNPs chr6:46616160_CTTTT/CT and rs3799866 in CYP39A1 are not present in the Table (compared to Table 1A) for not being significant. In **bold** are the SNPs that come significant when comparing GPD and HC patients as well. 1/2, etc. refers to genotypes 1/2, 1/3, 1/4, 1/5, 1/6, 2/3, 2/4, 2/5, 2/6, 3/4, 3/5, 3/6, 4/5, 4/6, 5/6; 0/2, etc. refers to genotypes 0/2, 0/3, 0/4, 0/5, 0/6.

	SNP	Genotype	OR	95% CI				*p*-value
				Lower	Upper			
**CYP46A1**	*rs4905883*	0/0	0.11	0.01		0.93	0.043	
	chr14:99722645_G/*	1/1	1.85	1.07		3.21	0.027	
	*chr14:99713865_CAAAAA/CAAAAAAA*	1/2, etc.	2.37	1.34		4.20	0.003	
**CYP7B1**	rs2356986	0/1	1.47	1.02		2.14	0.040	
	rs2356985	0/1	1.48	1.02		2.15	0.037	
	rs2884074	0/1	1.48	1.02		2.15	0.037	
	rs118111353	0/1	3.51	1.02		12.01	0.046	
	**rs16931331**	1/1	4.43	1.00		19.59	0.049	
	**rs16931334**	1/1	4.43	1.00		19.59	0.049	
	*chr8:64607398_TAAA/TA*	0/0	4.14	1.20		14.35	0.025	
	rs117732106	0/1	0.36	0.96		0.04	0.041	
	rs4477068	0/1	0.36	0.96		0.04	0.041	
**CYP39A1**	rs200377987	0/1	3.51	1.02		12.01	0.046	
	rs188027691	0/1	3.51	1.02		12.01	0.046	
	rs115993944	0/1	3.51	1.02		12.01	0.046	
	rs3799865	0/1	3.51	1.02		12.01	0.046	
	rs34289054	0/2, etc.	0.42	0.18		0.99	0.048	
	chr6:46650484_CTTTT/CTTTTTTT	1/1	0.36	0.16		0.80	0.013	
	*rs55887439*	0/0	1.92	1.16		3.17	0.011	
**CYP27A1**	**rs692303**	0/1	0.19	0.32		0.86	0.016	
	**rs73991002**	0/1	0.13	0.05		0.74	0.010	
	rs692258	0/1	0.22	0.03		0.61	0.031	
	rs692290	0/1	0.22	0.06		0.87	0.031	
	**rs74446825**	0/1	0.54	0.06		0.87	0.029	

### Overview of SNPs in the four cholesterol-degrading P450

Table 2 shows an overview of the number of all SNPs which are described in the PPMI data base being statistically significant in the four P450 genes when comparing IPD patients and controls (IPD/HC), GPD patients and HC (GPD/HC) as well as individuals showing a genetic predisposition and being with or without symptoms (GPD/GUN). CYP27A1 and CYP46A1 have rather low numbers of SNPs: only 475 and 734, respectively, as compared with 2,849 found in CYP7B1 and 1,700 found in CYP39A1. When analyzing the 24 SNPs in IPD patients, no SNP with OR>5 was described for the four P450, while two SNPs in CYP27A1 and one in CYP46A1 have been found which were clearly protective with OR<0.2. Focusing on SNPs which were either two to 5-fold over- (OR = 2.0–5.0) or under-represented (OR = 0.2–0.5) in IPD patients, in both cases three of the four P450 displayed such SNPs. The detailed summary of the statistics of SNPs in CYP46A1, CYP7B1, CYP39A1 and CYP27A1 given in Table 2 also demonstrates that in three out of the four P450 over-as well as under-represented SNPs occur in IPD patients. In contrast, CYP27A1 only displays SNPs which are under-represented indicating that SNPs in this gene might protect people from developing IPD. When looking at the OR values of the SNPs, CYP46A1 shows one SNP with the highest under-representation in IPD patients (OR = 0.11) followed by two SNPs in CYP27A1 with OR values of 0.13 and 0.19, respectively (Table 1B). On the other hand, SNPs with the highest over-representation are found in CYP7B1 (OR values of 4.43, 4.14 and 3.51) and CYP39A1 (OR values of 3.51) (Table 1B).

**TABLE 2:** Single nucleotide polymorphisms (SNPs) with statistically significant association (*p*-value <0.05) to Parkinson´s Disease (PD) for the genes of the four human cholesterol degrading cytochrome P450, CYP7B1, CYP27A1, CYP39A1 and CYP46A1. Compared are GPD and HC (GPD/HC), IPD and HC (IPD/HC) and GPD and GUN (GPD/GUN). SNPs for the various comparisons are subdivided into groups based on the strength of association to PD represented by the *odds ratio* (OR).

Substrate class	CYP	Total SNPs	OR < 0.2			OR = 0.2–0.5			0.5 < OR < 2.0			OR = 2.0–5.0					OR > 5		
			GPD/HC	IPD/HC	GPD/GUN	GPD/HC	IPD/HC	GPD/GUN	GPD/HC	IPD/HC	GPD/GUN	GPD/HC	IPD/HC		GPD/GUN		GPD/HC	IPD/HC	GPD/GUN
Sterols	7B1	2849	5	-	-	7	2	3	1	3	6	3	4		1		7	-	5
	27A1	475	2	2	-	5	2	-	-	1	15	-	-		-		-	-	-
	39A1	1700	3	-	-	24	2	6	89	1	4	74	4		4		1	-	2
	46A1	734	1	1	-	3	-	4	1	1	15	1	1		6		3	-	-

### Co-occurrence of associated SNPs

In a next step, we investigated the co-occurrence of associated SNPs in individual patients by analyzing the 24 SNPs identified as being statistically significant in the four P450 in all 362 IPD patients and in all 193 healthy controls available in the PPMI data base.

**FIGURE 2:**
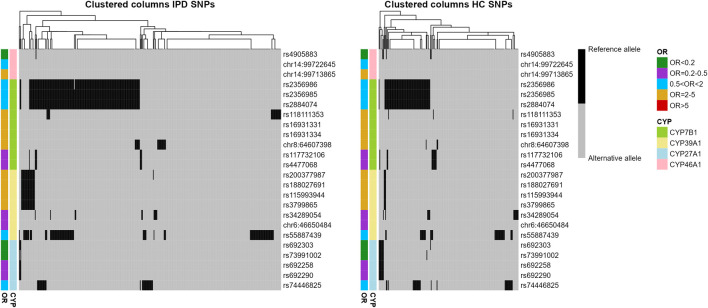
Distribution of reference and alternative alleles for 24 SNPs associated with IPD in CYP7B1, CYP39A1, CYP27A1 and CYP46A1. Binary heatmaps clustered hierarchically color-coding the allele for all IPD and HC samples (columns) and all 24 SNPs (rows). OR and P450 gene were color-coded and indicated per column and row.

Using the genotype information for the 24 SNPs from the four analyzed genes, a binary heatmap was constructed and hierarchically clustered for both IPD and HC samples (Table 1B; Figure 2). Interestingly, several genotypes tend to occur together, as, for example for rs2356986, rs2356985 and rs2884074 (all belong to CYP7B1 and are of moderate OR < 2). This subgroup forms a distinct cluster in some of the samples, in IPD as well as HC. In general, patients and controls tend to have more than one alteration in the same P450 gene.

Further analyzing the occurrence of SNPs in the corresponding P450, we observed that nearly 80% (79.3%) of the IPD patients had at least one PD-associated SNP in the CYP46A1, CYP7B1, CYP39A1 or CYP27A1 genes (75 out of 362 IPD patients did not show any associated SNP in these four genes) compared to 70% of HC (Table 3). When considering every cluster as one associated SNP, most patients (35.9%) had only one in one of the P450 genes while 24.9% had two and 14.6% had three associated SNPs (Table 3, column 5). A similar distribution was observed in HC (Table 3, column 9).

**TABLE 3:** Overview on the numbers of statistically significant SNPs in the four brain cholesterol degrading P450 in IPD patients and HC. The numbers and % of SNPs in all IPD patients (362) and HC (193), respectively, having a particular number of SNPs in the four P450 involved in cholesterol degradation (CYP7B1, CYP39A1, CYP27A1, CYP46A1) are shown. In columns 2, 3, six and seven every individual SNP is considered separately, whereas in columns 4, 5, eight and nine the SNPs arranged as a cluster (see Table 1B) are considered as one SNP.

Number of SNPs found in IPD patients	Number of IPD patients with the number of particular SNPs	% of IPD patients with the number of particular SNPs	Number of IPD patients with the number of particular SNPs (when every cluster is considered as one SNP)	% of IPD patients with the number of particular SNPs (when every cluster is considered as one SNP)	Number of HC individuals with the number of particular SNPs	% of HC individuals with the number of particular SNPs	Number of HC individuals with the number of particular SNPs (when every cluster is considered as one SNP)	% of HC individuals with the number of particular SNPs (when every cluster is considered as one SNP)
9 SNPs	3	0.8			1	0.5		
8 SNPs	6	1.7			0	0		
7 SNP	5	1.4			2	1.0		
6 SNPs	13	3.6			7	3.6		
5 SNPs	30	8.3	1	0.3	17	8.8	2	1.0
4 SNPs	63	17.4	13	3.6	21	10.9	7	3.6
3 SNPs	64	17.7	53	14.6	30	15.5	21	10.9
2 SNPs	28	7.7	90	24.9	17	8.81	36	18.6
1 SNP	75	20.7	130	35.9	40	20.7	69	35.8
0 SNPs	75	20.7	75	20.7	58	30.0	58	30.0

Another interesting question was whether the investigated patients displaying more than one associated SNP have these in different P450 or in the same one. Table 4 demonstrates that if we consider patients having SNPs only in one P450 and in no other of the four P450, the biggest number of patients have PD-associated SNPs in CYP7B1 (70 out of 362; 19.3% of all IPD patients) and the lowest number of patients have SNPs only in CYP27A1 (4/362; 1.1%). If we consider how many patients have associated SNPs simultaneously in two P450 from any combination of P450 and in no other of the four P450, the largest number of patients (41/362; 11.0%) have SNPs in both, CYP46A1 and CYP7B1, and the smallest number of patients (3/362; 0.83%) have SNPs in both, CYP39A1 and CYP27A1. However, 150 patients (41.4%) have SNPs either in CYP46A1 or in CYP7B1 or simultaneously in the two P450. Additionally, one patient (0.3%) had SNPs in all four P450, 31 patients (8.6%) had SNPs simultaneously in three P450, 109 patients (30.1%) in two P450, 146 patients (40.3%) in one P450 and 75 patients (20.7%) have SNPs in neither one of the four P450.

**TABLE 4:** Number of IPD patients having SNPs only in a certain P450 (CYP7B1, CYP39A1, CYP27A1, CYP46A1) and/or in a combination of two P450. The numbers for the combination of P450s display the numbers of SNPs found simultaneously in both or a distinct P450 from the combination (CYP46A1/CYP7B1; CYP46A1/CYP39A1; CYP46A1/CYP27A1; CYP7B1/CYP39A1; CYP7B1/CYP27A1; CYP39A1/CYP27A1) and in each P450 from the corresponding combination (CYP46A1 and CYP7B1; CYP46A1 and CYP39A1; CYP46A1 and CYP27A1; CYP7B1 and CYP39A1; CYP7B1 and CYP27A1; CYP39A1 and CYP27A1).

P450 or a combinations of P450	Number of IPD patients having SNPs only in the particular P450 or in the combination of P450	% of IPD patients having SNPs only in the particular P450 or a combination of P450
CYP46A1	39	10.8
CYP7B1	70	19.3
CYP39A1	33	9.1
CYP27A1	4	1.1
CYP46A1/CYP7B1	150	41.4
CYP46A1/CYP39A1	88	24.3
CYP46A1/CYP27A1	47	13.0
CYP7B1/CYP39A1	130	35.9
CYP7B1/CYP27A1	84	23.2
CYP39A1/CYP27A1	40	11.0
CYP46A1 and CYP7B1	41	11.3
CYP46A1 and CYP39A1	16	4.4
CYP46A1 and CYP27A1	5	1.4
CYP7B1 and CYP39A1	32	8.8
CYP7B1 and CYP27A1	11	3.0
CYP39A1 and CYP27A1	3	0.8

### Frequency of occurrence of associated SNPs

In a next step, the frequency of the 24 associated SNPs in IPD patients compared with the frequency in healthy controls (HC) was analyzed. Values of less than 5% to more than 40% for a corresponding SNP were observed (Table 5). When looking at CYP46A1, Table 5 shows that SNP rs4905883, present in an intron, is very rare. It is found in five out of 193 individuals of the HC group (2.6%) and in only one out of 362 IPD patients (0.3%) suggesting that this SNP protects people from developing IPD. In contrast, the two other SNPs, also present in introns of the CYP46A1 gene, can be found in 10% of HC and in 18% and 20%, respectively, of IPD patients. When analyzing SNPs in the CYP7B1 gene, two SNPs, occurring in a cluster and displaying a very high OR value of 4.43 (rs16931331 and rs16931334) are found in only two (1%) out of 193 HC but in 16 (4.4%) out of 362 IPD patients. Interestingly, the three SNPs of the second CYP7B1 gene cluster (rs2356986, rs2356985 and rs2884074) were found in 33% of HC and 42% of IPD patients. This suggests that these latter SNPs should have a less prominent effect on the manifestation of IPD, which is supported by an OR value of 1.48. A third cluster in CYP7B1 consisting of two SNPs (rs117732106 and rs4477068) is to be found in 7 (1.9%) IPD patients and in 10 (5%) HC. In CYP39A1, SNP rs55887439 is present in 16% of HC and 24.9% of IPD patients. The other 6 SNPs in CYP39A1 occur with a frequency of 1.55% in HC and 5.2% in IPD (the four clustered SNPs), 6.7% and 9.3% in HC and 3.0% and 3.6%, respectively, in IPD patients. As mentioned above, all SNPs in CYP27A1 are under-represented in IPD patients and mount between 3.6% and 14% in HC and 0.6% and 8% in IPD.

**TABLE 5:** Total number and percentage of individuals in the four groups (IPD, GPD, GUN and HC) having a particular SNP in one of the four cholesterol degrading P450 genes. When calculating OR values for every particular SNP, the genotype coming most abundant in healthy people was considered as the reference genotype. In **bold** the SNPs are shown that are significant when comparing HC and IPD patients, but also when comparing HC and GPD patients. The total number of IPD patients in the database is 362, that of GPD patients 317, that of GUN 344 and that of HC 193.

	SNP	Genotype	Number of individuals for IPD, GPD, GUN and HC having the SNP in the particular genotype	Number of individuals for IPD, GPD, GUN and HC having the SNP in the particular genotype in %
**CYP46A1**	rs4905883	0/0	1 IPD 4 GPD 1 GUN 5 HC	0.3 IPD 1.26 GPD 0.29 GUN 2.6 HC
	chr14:99722645_G/*	1/1	65 IPD 39 GPD 53 GUN 20 HC	18 IPD 12 GPD 15 GUN 10 HC
	chr14:99713865_CAAAAA/CAAAAAAA	1/2, etc.	74 IPD 47 GPD 54 GUN 20 HC	20 IPD 15 GPD 16 GUN 10 HC
**CYP7B1**	rs2356986	0/1	152 IPD 126 GPD 125 GUN 64 HC	42 IPD 40 GPD 36 GUN 33 HC
	rs2356985	0/1	153 IPD 126 GPD 125 GUN 64 HC	42 IPD 40 GPD 36 GUN 33 HC
	rs2884074	0/1	153 IPD 126 GPD 125 GUN 64 HC	42 IPD 40 GPD 36 GUN 33 HC
	rs118111353	0/1	19 IPD 1 GPD 6 GUN 3 HC	5.2 IPD 0.3 GPD 1.7 GUN 1.55 HC
	**rs16931331**	1/1	16 IPD 18 GPD 15 GUN 2 HC	4.4 IPD 5.7 GPD 4.3 GUN 1 HC
	**rs16931334**	1/1	16 IPD 18 GPD 15 GUN 2 HC	4.4 IPD 5.7 GPD 4.3 GUN 1 HC
	chr8:64607398_TAAA/TA	0/0	20 IPD 21 GPD 20 GUN 3 HC	5.5 IPD 6.6 GPD 5.8 GUN 1.55 HC
	rs117732106	0/1	7 IPD 12 GPD 16 GUN 10 HC	1.9 IPD 3.8 GPD 4.65 GUN 5 HC
	rs4477068	0/1	7 IPD 12 GPD 16 GUN 10 HC	1.9 IPD 3.8 GPD 4.65 GUN 5 HC
**CYP39A1**	rs200377987	0/1	19 IPD 12 GPD 15 GUN 3 HC	5.2 IPD 3.8 GPD 4.4 GUN 1.55 HC
	rs188027691	0/1	19 IPD 11 GPD 14 GUN 3 HC	5.2 IPD 3.5 GPD 4.1 GUN 1.55 HC
	rs115993944	0/1	19 IPD 12 GPD 14 GUN 3 HC	5.2 IPD 3.8 GPD 4.1 GUN 1.55 HC
	rs3799865	0/1	19 IPD 12 GPD 14 GUN 3 HC	5.2 IPD 3.8 GPD 4.1 GUN 1.55 HC
	rs34289054	0/2, etc.	11 IPD 10 GPD 18GUN 13 HC	3 IPD 3.2 GPD 5.2 GUN 6.7 HC
	chr6:46650484_CTTTT/CTTTTTTT	1/1	13 IPD 19 GPD 25 GUN 18 HC	3.6 IPD 6 GPD 7.3 GUN 9.3 HC
	rs55887439	0/0	90 IPD 69 GPD 71 GUN 31 HC	24.86 IPD 21.8 GPD 20.6 GUN 16 HC
**CYP27A1**	**rs692303**	0/1	3 IPD 4 GPD 2 GUN 8 HC	0.8 IPD 1.3 GPD 0.6 GUN 4 HC
	**rs73991002**	0/1	2 IPD 4 GPD 2 GUN 8 HC	0.6 IPD 1.3 GPD 0.6 GUN 4 HC
	rs692258	0/1	3 IPD 4 GPD 2 GUN 7 HC	0.8 IPD 1.3 GPD 0.6 GUN 3.6 HC
	rs692290	0/1	3 IPD 4 GPD 2 GUN 7 HC	0.8 IPD 1.3 GPD 0.6 GUN 3.6 HC
	**rs74446825**	0/1	29 IPD 22 GPD 20 GUN 27 HC	8 IPD 7 GPD 5.8 GUN 14 HC

### SNPs in relation to open chromatin, DNA methylation, and gene expression

As all of the 24 associated SNPs are within non-coding regions such as introns and 3′ untranslated regions of CYP7B1, CYP46A1, CYP39A1 and CYP27A1, they do not have a direct impact on protein structure. However, they could play an important role being localized within a regulatory region influencing expression of these genes. To examine such effects, we made use of a comprehensive dataset provided by the FOUNDIN-PD consortium. For 89 cell lines from the PPMI iPSC collection, we used available bulk ATAC-seq data from healthy controls, patients with IPD, and individuals carrying known disease-linked mutations and overlapped regions of open chromatin with the positions of the 24 SNPs of interest. Interestingly, two of these SNPs lie within open chromatin peaks of dopaminergic neurons after 65 days of differentiation (Table 6). SNP rs118111353 in CYP7B1 showed a peak for open chromatin in 29 samples (Table 6). Interestingly, all individuals underlying these samples showed the reference genotype. For SNP rs74446825 in CYP27A1 open chromatin was detected in four samples. Both SNPs are located in an intronic region.

**TABLE 6:** Number of individuals of PPMI cohort showing a peak of open chromatin at the genomic position of the 24 PD-associated SNPs or within a ±500 bp window around the SNP. Reference and alternate alleles are indicated. Two SNPs within or close to open chromatin for the majority of samples are highlighted in bold.

CYP	SNP	Chromosome	Location	Number of samples in open chromatin	Number of samples within ±500 bp of open chromatin	Reference	Alternative	Variant location
CYP46A1	rs4905883	chr14	99719392	0	0	G	T	Intron
	chr14:99722645_G/*	chr14	99722645	0	0	G	GTGTGTGTGTC	Intron
	chr14:99713865_CAAAAA/CAAAAAAA	chr14	99713865	0	0	CAAAAA	CAAAAAAA CAAAAAAAAA CA,C,CAAAA CAAAAAA	Intron
CYP7B1	rs2356986	chr8	64701869	0	5	C	T	Intron
	rs2356985	chr8	64701977	0	4	C	G	Intron
	rs2884074	chr8	64702184	0	0	G	T	Intron
	**rs11811** **1** **353**	chr8	64726077	29	45	T	C	Intron
	rs16931331	chr8	64591473	0	0	A	G	3′ UTR
	rs16931334	chr8	64591813	0	0	C	T,A	3′ UTR
	chr8:64607398_TAAA/TA	chr8	64607398	0	3	TAAA	TA,TAA,TAAAA,T	Intron
	rs117732106	chr8	64656091	0	0	C	G	Intron
	rs4477068	chr8	64670875	0	0	T	G	Intron
CYP39A1	rs200377987	chr6	46609103	0	0	G	GA	Intron
	rs188027691	chr6	46611705	0	0	C	A	Intron
	rs115993944	chr6	46628780	0	1	C	T	Intron
	rs3799865	chr6	46629771	0	1	T	C	Intron
	rs34289054	chr6	46613939	0	0	CA	C,AA,CAA	Intron
	chr6:46616160_CTTTT/CT	chr6	46616160	0	0	CTTTT	CT,C	Intron
	chr6:46650484_CTTTT/CTTTTTTT	chr6	46650484	0	0	CTTTT	CTTTTTTT, CTTTTT CTTTTTT,C CTTTTTTTT,CT	Intron/upstream gene
	rs3799866	chr6	46628549	0	0	A	C	Intron
	rs55887439	chr6	46634524	0	0	CT	TT,C,CTT	Intron
CYP27A1	rs692303	chr2	2.19E+08	0	0	C	T	Intron
	rs73991002	chr2	2.19E+08	0	0	A	C	Intron
	rs692258	chr2	2.19E+08	0	0	C	T	Intron
	rs692290	chr2	2.19E+08	0	0	G	T	Intron
	**rs74446825**	chr2	2.19E+08	4	50	G	A	Intron

Next, we evaluated DNA methylation around the SNPs. While none of the 24 SNPs was found directly within a CpG island, four SNPs (rs4905883, rs692303, rs692290, rs74446825) were within a 500 bp window from CpG site on the EPIC array. However, DNA methylation levels of these CpG sites did not significantly differ between healthy controls and patients and showed no clear correlation with the genotype of the SNP.

**FIGURE 3:**
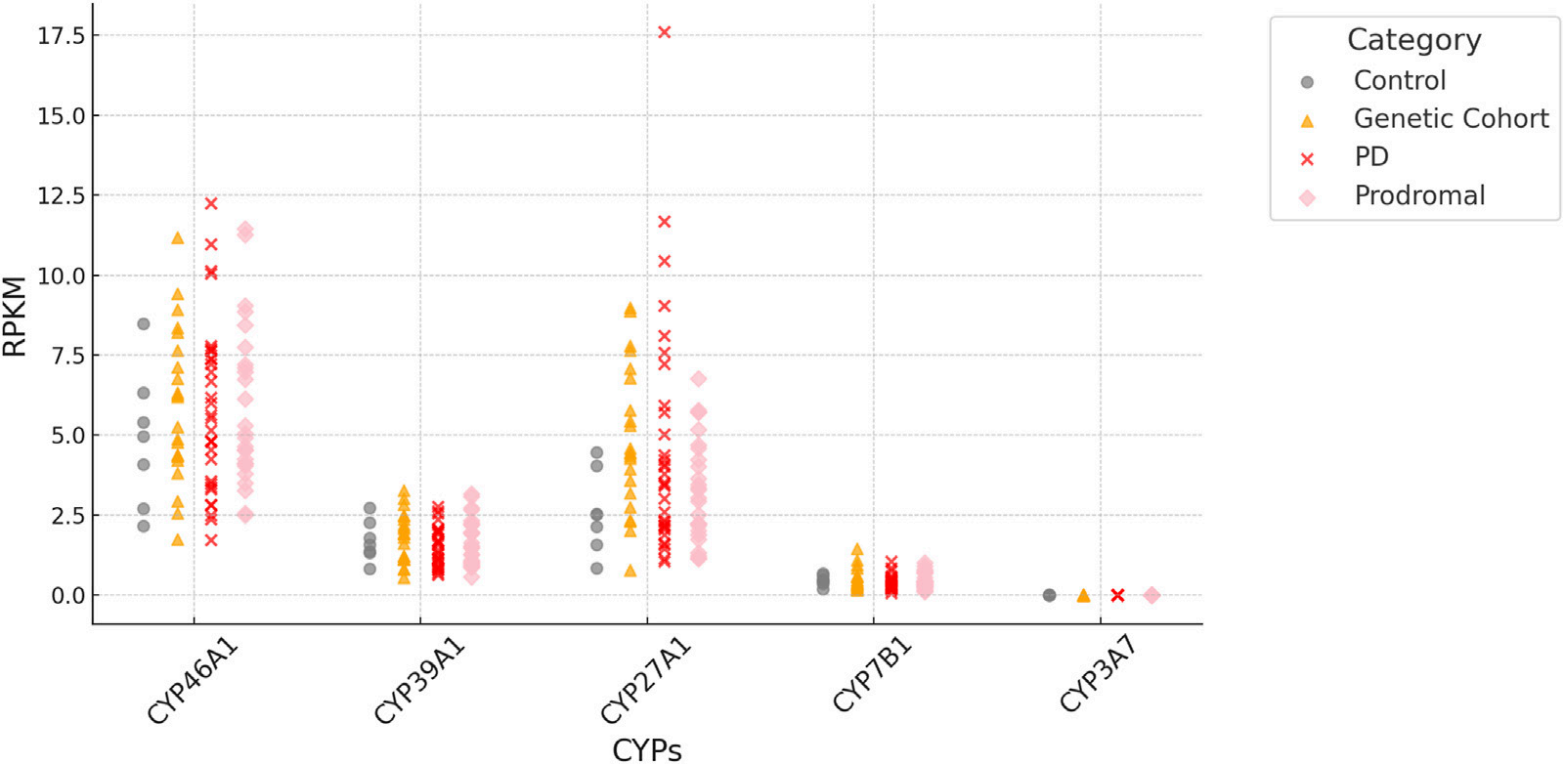
RPKM (Reads Per Kilobase per Million mapped reads) of *CYP7B1*, *CYP46A1*, *CYP39A1* and *CYP27A1* based on bulk RNA-seq data of iPSC-derived neurons at day 65. Values are based on individual samples from FOUNDIN-PD bulk RNA-seq data sets and shown for healthy controls as well as PD patients (genetic cohort, PD, prodromal). The expression of CYP3A7 is shown as control.

**FIGURE 4:**
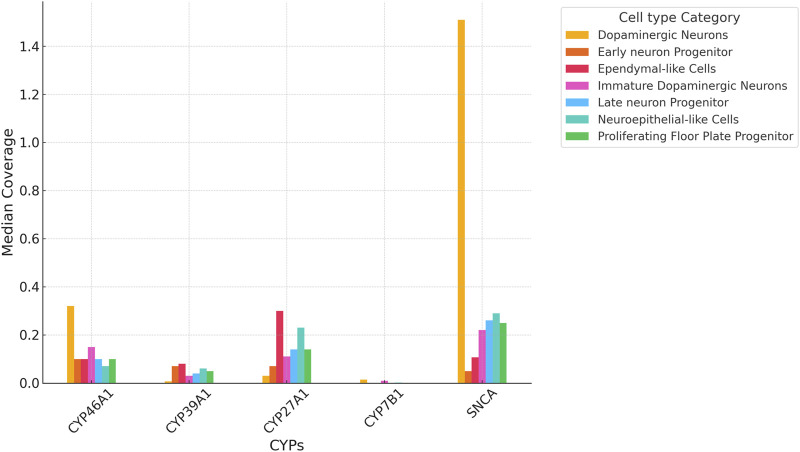
Median coverage of *CYP7B1*, *CYP46A1*, *CYP39A1* and *CYP27A1* based on single cell RNA-seq data of iPSC-derived neurons at day 65. Values compare expression levels in individual cell types and are based on values from the FOUNDIN-PD explorer (https://www.foundinpd.org/#Foundinpd). The expression of α-synuclein (SNCA) is shown as control.

Importantly, all four P450 genes, CYP7B1, CYP46A1, CYP39A1 and CYP27A1 are expressed in iPSC-derived neurons at day 65 from the FOUNDIN-PD cohort with strongest expression of CYP46A1 and CYP27A1 (Figure 3). In comparison, CYP3A7 which is known to be preferentially expressed in fetal liver, shows no expression under these conditions. Overall, there are only minor expression differences of CYP7B1, CYP46A1, CYP39A1 and CYP27A1 in PD patients *versus* healthy controls. Comparing the median coverage of these genes in single cell RNA-seq data, indicated strongest expression of CYP46A1 in dopaminergic neurons similar to SNCA, and CYP27A1 in ependymal-like cells (Figure 4). Taken together, these expression data indicate that mainly CYP46A1 might play an important role in dopaminergic neurons and the context of Parkinson’s disease.

## Discussion

In this manuscript, we present evidence that SNPs in cholesterol-degrading P450 genes play a role in PD and, hence, extend the notion that cholesterol plays an important role in the brain and may be related to the development of PD (reviewed by (Jin et al., 2019) and (Alrouji et al., 2023)). Based on the observation that cholesterol is of importance in the CNS and that a changed turnover across the brain is involved in neurodegenerative disorders such as Alzheimer’s disease and Niemann-Pick type C disease (reviewed by (Dietschy and Turley, 2001)), cholesterol metabolism and turnover came into the focus of PD as well (Dietschy and Turley, 2004). To date the association is still debated since cholesterol metabolism in the CNS might not reflect the situation in the periphery and not enough data are available to draw conclusions about an association between cholesterol metabolism and PD. A bidirectional effect of cholesterol was described via deregulation of ion channels and receptors playing a neuroprotective role as well as induction of oxidative stress and inflammation. More recently, binding of cholesterol to α-synuclein and its role in the formation of oligomers and fibrils have been investigated (Zhaliazka et al., 2024). However, so far, a broader view on changes in the metabolism of cholesterol and possible consequences for the etiology of PD is missing and thus became the focus of our investigations.

Based on our initial studies (Hartz et al., 2022; Petkova-Kirova et al., 2023), here we performed a detailed analysis of all statistically significant SNPs in the four P450 involved in cholesterol degradation in IPD patients compared with HC. Analyzing the effect of SNPs in the four individual P450 genes, it first becomes obvious that CYP27A1 and CYP46A1 have a smaller number of significant as well as total SNPs compared to CYP7B1 and CYP39A1. One of the reasons may be the size of the genes which is about 33.6 kb, 43.0 kB, 212.2 kB and 103.3 kB, respectively (based on GeneCards).

As mentioned in (Hartz et al., 2022), some reference alleles of SNPs in the used reference genome did not reflect the reference but the alternate allele. We, therefore, thoroughly investigated all SNPs observed in the four P450 under study and corrected them for such mistakes focusing on IPD patients. Although all 24 significant SNPs can also be found in HC, approximately 80% of IPD patients display SNPs at least in one of the four genes involved in cholesterol degradation (Table 3), while in HC only 70% of individuals have SNPs in one of the four P450 genes. This suggests a role of the cholesterol-degrading P450 genes in the etiology of IPD. The fact that the SNPs observed in IPD patients also occur (although to a lower extent) in HC indicates that the corresponding SNP alone is not sufficient for causing IPD and that PD most likely is a polygenic/multifactorial condition. This is supported by our previous study, where we reported that additional SNPs in P450 genes shift individuals with a predisposition but without symptoms (GUN) to a disease state (GPD patients) (Petkova-Kirova et al., 2023). Similar data were also published for beta-glucocerebrosidase (GBA), mutations of which are the most common genetic risk factor for PD (Neumann et al., 2009; Ran et al., 2022). Mutations of GBA were not only found in PD patients but also in healthy controls (10.1% vs*.* 3.8%) (Muldmaa et al., 2021). Analyses of mutants in GBA from 16 centers detected two SNPs with OR values of 3.96 (for mutant N370S) and 6.73 (for mutant L444P) (Sidransky et al., 2009). Among Ashkenazi Jewish individuals, either mutation was found in 15% of patients and 3% of controls, whereas among non-Ashkenazi Jewish individuals either mutation was found in 3% of patients and less than 1% of controls. As shown in Table 5, similar ratios were observed for SNPs in three of the four P450 genes (CYP27A1 only has SNPs with OR values < 1) when comparing IPD and HC individuals. However, while patients with GBA mutations present earlier with the disease, this was not observed in IPD patients with SNPs in the cholesterol-degrading P450 genes of CYP7B1, CYP39A1 and CYP46A1, independent of the number of SNPs found (1, two or more). Further studies on the occurrence and the consequences of the SNPs in CYP7B1, CYP39A1 and CYP46A1 using other data bases as well as clinical results are necessary to test whether those P450 may also be classified as possible genetic risk factors for the pathology of PD as is GBA. In contrast, CYP27A1 only displayed SNPs with OR values < 1, suggesting that the corresponding SNPs are protective. It has, however, to be taken into account that CYP27A1 displays effects on various pathways. Besides catalyzing the conversion of cholesterol into 27-hydroxycholesterol, it is also involved in reactions leading to bile acids as well as in the biosynthesis of active vitamin D catalyzing the hydroxylation of vitamin D to form 25-hydroxy vitamin D. Interestingly, two SNPs of CYP7B1 (coming clustered and of the highest OR value among the 24 SNPs (OR = 4.43)) and three SNPs of CYP27A1 are also found with statistical significance in GPD patients compared with HC (Table 1B in bold). This underlines their role as possible players in the etiology of PD.

The vast majority of SNPs associated with diseases map to the noncoding part of the genome and are often found in introns and intergenic regions. To understand the potential impact of these SNPs it is important to evaluate whether they are found in a regulatory region and hence may affect gene expression. One mechanism how such a SNP can affect expression is the alteration in the transcription factor binding via creation or disruption of transcription factor binding sites (TFBSs). For the 24 SNPs, we checked whether open chromatin around the SNP indicates a regulatory region. Many TFBS are only based on predictions and open chromatin can be a good approximation for regulatory regions. Hence, ATAC-seq data from FOUNDIN-PD were a great resource to evaluate the 24 SNPs and, indeed, we found two of them to be in open chromatin. When analyzing ATAC-seq data, SNPs rs118111353 (CYP7B1) and rs74446825 (CYP27A1) are found to be located directly in open chromatin (Table 6) indicating a possible role of the two SNPs in the etiology of PD. The latter SNP is of special importance as it comes significant when comparing GPD patients and healthy controls as well (Table 1B in bold). Furthermore, there is a methylation site, CpG (cg02930667) at 175 bp away from the SNP.

Although there are (besides our results) to the best of our knowledge no direct indications in the literature that SNPs in the P450 involved in the degradation of cholesterol are associated with PD, many indirect pieces of evidence exist, which underpin our conclusions. The contribution of CYP46A1 to various neurological diseases and potential ways to regulate the activity of this enzyme is discussed in a recent review (Pikuleva and Cartier, 2021). Moreover, CYP46A1 has been supposed to be involved in the etiology of PD by studying the plasma and cerebrospinal fluid (CSF) levels of 24-hydroxycholesterol in patients with PD (Bjorkhem et al., 2013). It was found that there was a significant correlation between levels of 24-hydroxycholesterol in CSF and the duration of PD indicating a role of CYP46A1 which catalyzes the conversion of cholesterol to 24-hydroxycholesterol. Neurodegeneration results in an increased efflux of 24-hydroxycholesterol from neurons into CSF. However, due to a disruption of the blood-brain barrier and reduced capacity of the neuronal enzyme CYP7B1 to metabolize 27-hydroxycholesterol, 27-hydroxycholesterol is also increased in CSF upon neurodegeneration (Bjorkhem et al., 2018). A very recent study investigating 60 PD patients and 64 controls demonstrated that higher levels of plasma 24-hydroxycholesterol are inversely associated with PD risk (Huang et al., 2019). While 24-hydroxycholesterol is formed in neurons, 27-hydroxycholesterol is mainly formed in extracerebral tissues and organs (although the ubiquitous CYP27A1 is also expressed in brain), but continuously taken up from the circulation into the brain. Knock-out mice lacking CYP27A1 or CYP46A1 demonstrated that cholesterol synthesis was suppressed in *Cyp46a1* ^*−/−*^ but not in *Cyp27a1* ^*−/−*^ mice indicating a critical role of CYP46A1 in cholesterol efflux and metabolism (Xie et al., 2003). Moreover, a stable expression of cerebral CYP46A1 was demonstrated indicating a housekeeping function, while the expression of CYP27A1 may vary (Mast et al., 2010).

After an initial hydroxylation of cholesterol by CYP46A1 or CYP27A1, a second hydroxylation by CYP39A1 and by CYP7B1, respectively, takes place to further convert cholesterol into precursors of bile acids. Both enzymes, CYP39A1 and CYP7B1, catalyze a hydroxylation in 7α-position using either 27- or 24-hydroxycholesterol as substrate (Figure 1). Low expression of the corresponding genes of these two P450 would lead to an increase in 24- or 27-hydroxycholesterol in the brain. While there are no direct data on an association of CYP39A1 SNPs and PD, four intronic SNPs of CYP39A1 are associated with the occurrence of motor fluctuations 5 years after the onset of PD (Ryu et al., 2020). Unfortunately, no explanation concerning functional consequences of these SNPs was given (Ryu et al., 2020). The described SNPs are different from the ones found to be significant in our studies. Investigating the expression of the CYP39A1 gene, it was described that the nuclear receptor RORα regulates CYP39A1 expression levels in human hepatoma cells. Binding of the RORα receptor was located upstream of the promoter and downstream of the first intronic region of the CYP39A1 transcription start site, resulting in CYP39A1 expression regulation (Matsuoka et al., 2020).

Interestingly, when combining metabolomics and epigenetics, the primary pathway of bile acid biosynthesis (including the reactions catalyzed by CYP46A1 and CYP39A1) has been shown to be perturbed in PD patients and a methylation locus was found in the promotor region of CYP39A1, possibly suppressing its expression (Vishweswaraiah et al., 2022).

The other enzyme catalyzing a 7α-hydroxylation of hydroxy-cholesterol and this way producing another precursor of bile acid synthesis, is CYP7B1. It uses 27-hydroxycholesterol as substrate (Figure 1). We found 9 SNPs displaying a significant over- or under-representation in IPD patients compared with HC. The SNP cluster consisting of rs16931331 and rs16931334 is highly over-represented not only in IPD patients (IPD vs. HC, OR = 4.43), but also in GPD patients (GPD vs. HC, OR = 6.36) supporting its role in the etiology of PD (Hartz et al., 2022). SNP rs118111353 might also be considered to significantly contribute to IPD. Except for displaying one of the highest ORs (OR = 3.51) when IPD and HC are compared, this SNP is also over-represented in IPD patients compared to GUN and especially IPD compared to GPD patients (IPD vs. GPD, OR = 17.5; IPD vs. GUN, OR = 3.12). Further analysis using the data from the 89 individuals in the ATAC-seq study demonstrated that SNP rs118111353 of CYP7B1 is located exactly within open chromatin in 29 individuals and in 16 more individuals at a distance of ±500 bp from open chromatin. Most interestingly, the 45 individuals (29 + 16) showing the location of the SNP within ±500 bp of open chromatin have a 0/0 genotype. This genotype is also found in regions of closed chromatin. Genotype 0/1 is very rare for this SNP and only present in very few individuals of the PPMI data base. Two of them are found in the FOUNDIN-PD data base as well. These two individuals are IPD patients and have no open chromatin in close proximity of the SNP. This may be explained by the following alternatives: (i) it indicates that most probably in the presence of the SNP opening of chromatin is prevented; (ii) since there are only two individuals found with a genotype of 0/1, the presence of this SNP in a region of chromatin far away from open chromatin is statistically not supported. Further investigations are necessary to find the correct explanation.

SNPs in the CYP7B1 gene leading to a dysfunction of this enzyme were shown to cause increased levels of 25- and 27-hydroxycholesterol in the plasma of affected individuals. This was also shown to lead to another neurological disease, spastic paraplegia type 5 (Stiles et al., 2009). Moreover, 7α,27-dihydoxycholesterol was shown to modulate the immune response (inflammatory processes are key factors in the etiology of PD) (Soroosh et al., 2014).

Considering the effect of each P450 gene on the association with IPD, it also has to be taken into account which of the two pathways (CYP46A1/CYP39A1 or CY27A1/CYP7B1) is contributing most to the degradation of cholesterol from brain (forming precursors of bile acids) and to PD. It was shown that CYP46A1 is responsible for the majority of cholesterol turnover in the brain (Lutjohann et al., 1996; Russell et al., 2009). In the FOUNDIN-PD data, we found CYP46A1 to be strongly expressed with the largest coverage in dopaminergic neurons (Figures 3, 4). Nevertheless, SNPs in CYP7B1 also seem to play an important role for the association with IPD (Table 4). The importance of cholesterol degradation for the etiology of PD is further supported by results indicating an effect of bile acids, products of cholesterol conversion, in the manifestation of PD (Li et al., 2021). It was demonstrated that individuals suffering from PD have biliary abnormalities. This way very complex networks may occur when SNPs in the four cholesterol-degrading P450 are associated with PD, since not only the immediate effect of the changed oxysterol metabolite has to be taken into account, but also the effect on the formation of downstream metabolites like bile acids and their effect on the development of PD.

A very important question is whether the data found here may have relevance for novel (and more causative) treatments of PD patients. There are conflicting results concerning the association between serum cholesterol and PD, since it is well-known that cholesterol metabolism in the CNS is independent on the one in the periphery. However, high levels of cholesterol in the brain seem to aggravate PD (Jin et al., 2019). Statins have been discussed as potential drugs to treat PD, since they reduce neuronal α-synuclein aggregation in *in-vitro* models (Bar-On et al., 2008). Results based on the analysis of 64 PD patients and corresponding controls recommend statin usage for PD patients but also emphasize the need for further research in this field (Huang et al., 2019). Recently, liver X receptors (LXRs) were shown to play a critical role in PD by inhibiting neuroinflammation due to their effect on brain cholesterol homeostasis and thus might indicate a novel approach for treating PD (Alnaaim et al., 2024). In addition, it has to be taken into account that the SNPs found here to be associated with the occurrence of IPD are part of a polygenetic and/or multifactorial nature of the disease. Thus, a combination of various factors (e.g. occurrence of SNPs in different genes or combination of a SNP with environmental or physiological factors) instead of mutations in a single gene may be responsible for the expression of the disease symptoms (Hartz et al., 2022; Petkova-Kirova et al., 2023; Bogers et al., 2023). Nevertheless, knowing the association of a certain SNP in the cholesterol degrading P450 with the occurrence of IPD, can be used as a basis for a personalized and causative treatment of the disease. Looking at CYP46A1, the main enzyme responsible for cholesterol degradation in the brain, it turns out that disturbed expression or function of this enzyme will lead to an increase of cholesterol in the brain and a decrease of its turnover to finally produce bile acids. Such a defect has also been described as one of the factors associated with Alzheimer’s disease and approaches to treat this disease have been developed (Mast et al., 2014; Pikuleva, 2021). In these studies efavirenz turned out to be especially promising (Mast et al., 2014; Pikuleva, 2021; Mast et al., 2024). Interestingly, artificial intelligence-driven drug repositioning combined with screening of a compound library indicated a potential effect of efavirenz on PD pathology as well (Kim et al., 2024). Concerning SNPs in CYP27A1, the other cholesterol-hydroxylating enzyme, a pleiotropic effect can be expected due to its additional involvement in vitamin D and bile acid biosynthesis. The product of the CYP27A1 reaction, 27-hydroxycholesterol, is considered to increase oxidative stress and to induce Alzheimer’s disease as well as PD (Marwarha and Ghribi, 2015). This observation coincides with our data showing an under-representation of the five SNPs of CYP27A1 in IPD patients as well as some of them in GUN and GPD patients (for example, rs74446825, rs692303 and rs73991002 are under-represented in GPD patients as well). An increase in 27-hydroxycholesterol also accelerates the aggregation of α-synuclein (Dai et al., 2023) and might be observed when the function of CYP7B1 is disturbed. Therefore, selective inhibition of CYP27A1 may be a promising tool to treat patients suffering from this special type of PD. On the other hand, the product of the CYP7B1 reaction (7α, 27-dihydroxycholesterol, see Figure 1), was shown to reduce the number of midbrain dopamine neurons. This suggests that selective inhibitors of CYP7B1 might be useful for PD therapy in this case. Voriconazole has been demonstrated to prevent the loss of these neurons (Hennegan et al., 2024), but more selective inhibitors may be developed for this treatment. Much research has been performed in this field and potential selective inhibitors have already been developed for different P450 (Schuster and Bernhardt, 2007). Finally, CYP39A1, catalyzing the conversion of 24-hydroxycholesterol to 7α, 24-dihydroxycholesterol, was shown to be regulated by the orphan nuclear receptor RORα. This knowledge can also be used to develop a personalized treatment involving modulators of the corresponding receptor and to regulate in this way the activity of CYP39A1 to stop accumulation of 24-hydrxycholesterol (Matsuoka et al., 2020).

Taken together, our results demonstrate that cholesterol and the metabolites of cholesterol degradation display a multitude of effects on the development of PD and that the four P450 involved in the degradation of cholesterol and production of bile acid precursors are crucial for this process. Some of the SNPs were shown to be located within open chromatin or in close distance ( ± 500bp) to open chromatin (CYP7B1, CYP27A1). Limitations of our study are, first, that statistical analysis based on a limited set of data (such as, for example the PPMI data base) may always generate false negative or false positive results and affect the characterization of individual SNPs. Due to the focus on the PPMI data base, a limited number of patients and HC was investigated and the influence of ethnicity and other factors on the results was not studied. This limitation is, however, common to most of the identified PD risk factors, where only very few GWAS loci have been systematically assessed so far. Second, limited data are available on the functional consequences of the observed SNPs although we investigated the location of the identified SNPs in open chromatin and potential methylation sites. Moreover, data on the effect of the SNPs on regulatory functions are very difficult to obtain as common for such studies. The ATAC-seq data are based only on 1 cell type - dopamine neurons. One might miss regulatory potential present, for example, in glial cells. Other resources would need to be investigated. Nevertheless, analyzing the defects in the four P450 involved in cholesterol degradation in IPD in corresponding patients may be useful to develop a personalized approach for the treatment of this disease.

## Data Availability

Publicly available datasets were analyzed in this study. This data can be found here: https://www.ppmi-info.org/access-data-specimens/download-data.

## Abbreviations

CYP, cytochrome P450; P450, cytochrome P450; Adx, adrenodoxin; POR, NADPH-dependent cytochrome P450 reductase; PD, Parkinson’s disease; IPD, idiopathic (sporadic) PD; GPD, PD with a genetic predisposition; GUN, persons with genetic predisposition for PD, but without symptoms of disease; PPMI, The Parkinson’s Progression Markers Initiative; OR, odds ratio; CI, confidence interval.
